# Characterization of CTC-HER2 Status in Patients with HER2-Low Invasive Breast Cancer

**DOI:** 10.3390/cancers18203265

**Published:** 2026-10-10

**Authors:** Jennifer H. Chen, Salyna Meas, Roland L. Bassett, Vanessa N. Sarli, Joshua Upshaw, Anthony Lucci

**Affiliations:** 1 Department of Breast Surgical Oncology, University of Texas MD Anderson Cancer Center, 1400 Pressler Street, Unit 1484, Houston, TX 77030, USA; jennifer.chen2@bcm.edu (J.H.C.);; 2 Department of Biostatistics, University of Texas MD Anderson Cancer Center, 7007 Bertner Avenue, Unit 1689, Houston, TX 77030, USA

**Keywords:** HER2 status, HER2-low breast cancer, circulating tumor cells, CTC-HER2, liquid biopsy

## Abstract

**Background:** HER2 status is a dynamic process where one in ten patients with invasive breast cancer may undergo receptor conversion. We characterized HER2 status on circulating tumor cells (CTCs) in patients with HER2-low breast cancer. **Methods:** Patients with HER2-low expression, defined as immunohistochemistry (IHC) score of 1+ or 2+ with negative HER2 fluorescent in situ hybridization, were enrolled from 2019 to 2023. All patients were treatment-naïve to HER2-targeted therapies. Blood draws were collected serially q3–6 months. CTCs were enumerated using CellSearch™ and HER2 expression on CTCs were detected using an anti-HER2 fluorescent antibody. Presence of ≥1 CTCs was considered positive. Wilcoxon rank-sum, Fisher’s exact test, and Kaplan–Meier method were used for statistical analysis. **Results:** Among 39 HER2-low patients, median age was 49.0 years (IQR 40.5–58.5). Patients were predominantly Non-Hispanic White (26, 66.7%), with node-positive (29/38, 76.3%), ductal (27, 69.2%), stage II-III (24, 61.5%) disease. Over half (23, 59.0%) had inflammatory breast cancer (IBC). Rates of ER and PR positivity were 56.4% (22) and 51.3% (20), respectively. Majority of patients underwent surgery (23 mastectomy, seven segmental mastectomy) with a pCR rate of 17.2%. Up to 53.8% (21) of patients had HER2-positive CTCs during treatment course. Of these, 13 (61.9%) and eight (38.1%) were present before and after surgery, respectively. There were no differences in age, BMI, grade, histology, lymphovascular invasion, IBC status, and pCR rate by CTC-HER2 status. At a median follow up of 3.8 years (95% CI: 2.6–4.9), patients with HER2-positive CTCs demonstrated a trend of worse prognosis compared to those with HER2-negative CTCs (5-year overall survival 59.8% vs. 88.9%, *p* = 0.11). **Conclusions:** In this study cohort, a high proportion of HER2-low expressors demonstrated HER2-CTC positivity. These findings support the potential use of HER2 expression on CTCs as a biomarker to personalize therapies among patients with HER2-low breast cancer.

## Simple Summary

In this prospective cohort study of patients with HER2-low invasive breast cancer, characterization of HER2 status on circulating tumor cells enumerated from serial blood draws demonstrated wide heterogeneity in HER2 expression. Among 39 patients, rate of CTC-HER2 expression was over 50% despite low HER2 expression on the primary tumor. Importantly, variation in expression occurred both within and across different timepoints, further highlighting the dynamic nature of HER2 expression. Survival analysis demonstrated a trend toward worse overall survival among patients expressing CTC-HER2 compared to those without HER2 expression. These exploratory results highlight the potential use of CTC-HER2 status in larger cohort studies evaluating HER2-targeted treatment selection and subsequent impact on survival analyses.

## 1. Introduction

In recent years, the definition of HER2 (human epidermal growth factor receptor 2) receptor has evolved substantially [1]. While its classification has traditionally been categorized as a dichotomous variable, studies suggest its expression actually exists along a dynamic spectrum [2]. The original criteria for HER2-positivity, which included protein overexpression measured by immunohistochemistry (IHC) and gene amplification measured by fluorescent in situ hybridization (FISH), have expanded to include new classifications such as HER2-low disease [3]. HER2-low breast cancer has been shown to encompass up to 60% of previously “HER2 negative” cases, where HER2 expression is not overexpressed or amplified but remains detectable nonetheless [1,3].

The significant heterogeneity in HER2 expression is further magnified by the high discordance rates in HER2 expression between the primary breast tumor and metastatic site(s) [2,4,5]. Studies cite that among metastatic HER2-negative disease, up to 50% may exhibit discrepancy in HER2 receptor status between the primary tumor versus metastatic site(s) [2,4]. Furthermore, characterization of HER2 on circulating tumor cells (CTC), cancer cells shed into the bloodstream from the primary tumor or sites of metastasis, have presented another avenue to assess HER2 status and track receptor status in real time [6,7,8,9,10,11]. While prior studies on HER2 dynamics have focused exclusively on HER2-negative breast cancer or all subtypes of invasive breast cancer combined, similar investigations in HER2-low breast cancer in the era of new HER2 classification are lacking. Given that recent studies have demonstrated a survival benefit with HER2-targeted antibody drug conjugates in HER2-low breast cancer, accurate evaluation of HER2 status remains imperative to inform future treatment decision making [12,13].

The objective of this exploratory study was to evaluate HER2 expression among patients with HER2-low invasive breast cancer at a microscopic level through enumeration and characterization of circulating tumor cells. We hypothesized that a high proportion of patients with HER2-low disease would express HER2 on their CTCs. Specifically, our aims were (1) to determine the prevalence of HER2 expression on CTCs among patients with HER2-low breast cancer and (2) to evaluate the association of HER2 expression with overall survival.

## 2. Methods

### 2.1. Study Design

This was a prospective registry-based cohort study of patients with invasive breast cancer and HER2-low disease treated at a large comprehensive cancer center from 2019 to 2023 (The University of Texas MD Anderson Cancer Center, Houston, TX, USA). Inclusion criteria included any patient with biopsy-confirmed invasive breast cancer, including inflammatory breast cancer (IBC), with available CTC-HER2 assessment during study enrollment. Exclusion criteria included patients with prior treatment and/or recurrent disease. Based on American Joint Committee on Cancer (AJCC) 8th edition guidelines, IBC was defined as (1) rapid onset of breast erythema or edema within six months, (2) erythema involving at least one-third of breast, and (3) biopsy-confirmed presence of invasive carcinoma [14]. Patients underwent serial blood draws throughout treatment course every 3–6 months. Treatments included neoadjuvant systemic therapy and surgical resection, with or without adjuvant radiation therapy in the event of breast conserving surgery receipt. Systemic therapy and radiation therapy were determined by the treating medical oncologist and radiation oncologist, respectively. No patients received HER2-targeted therapies. Pathologic complete response (pCR) was defined as the absence of invasive tumor in the breast and axillary lymph nodes in the surgical specimen. Timepoints for CTC assessments consisted of baseline pretreatment, during neoadjuvant therapy, post-neoadjuvant therapy but before surgery, and post-operative follow up every 3–6 months. Informed consent was obtained from all patients prior to study enrollment. This study was approved by the Institutional Review Board at the University of Texas MD Anderson Cancer Center (Houston, TX, USA).

### 2.2. Primary Tumor HER2 Status

Primary tumor HER2 status was determined using a combination of IHC and FISH per institutional protocol. HER2 protein expression on primary tumor was graded from 0 to 3+ (HER2 Roche Tissue Diagnostics, 4B5 rabbit monoclonal antibody, Tucson, AZ, USA). All patients with IHC score of 2+ underwent FISH testing. HER2-low disease was defined as patients with IHC score of 1+ or IHC score of 2+ with negative HER2 FISH. HER2 positivity was defined as patients with IHC score of 3+ or IHC score of 2+ with positive HER2 FISH. HER2 negativity was defined as patients with IHC score of 0.

### 2.3. CTC Detection

Circulating tumor cells were quantified using CellSearch (Menarini Silicon Biosystems, Huntingdon Valley, PA, USA), an FDA-cleared method for detection and isolation of CTCs from peripheral blood through a series of negative and positive immunomagnetic enrichment steps. Blood samples containing a minimum of 7.5 mL of peripheral venous blood per patient were collected using 10 mL CellSave tubes. Samples underwent positive enrichment through binding to anti-EpCAM (epithelial cell adhesion molecule) antibodies bound to ferrofluid nanoparticles. Cells were then stained with DAPI (4′,2-diamidino-2-phenylindole) to identify nucleated cells, anti-cytokeratin antibodies (CK8, CK18, CK19) to identify epithelial cytokeratin proteins, and anti-CD45 antibodies (leukocyte marker) to select out circulating white blood cells. CTCs (DAPI+, CK 8/18/19+, and CD45-) were identified under direct visualization using the CellTracks Analyzer, a semi-automated fluorescent microscope. Presence of 1 or more CTCs was considered positive.

### 2.4. CTC-HER2 Detection

HER2 status on CTCs was assessed using an anti-HER2 fluorescent antibody (CellSearch Tumor Phenotyping Reagent Anti-HER2-FLU, Menarini Silicon Biosystems, Huntingdon Valley, PA, USA) using manufacturer instructions and established published protocol [15,16]. Samples with detectable CTCs were eligible for CTC-HER2 expression evaluation. HER2 status was assessed based on the presence or absence of any HER2 immunofluorescent signal. Any CTC that demonstrated detectable HER2 signal was classified as CTC-HER2 positive. CTCs that did not exhibit a HER2 signal were classified as CTC-HER2 negative. Due to small sample size, HER2 expression was not scored individually by IHC intensity (1 to 3+). All sample preparations and evaluations of CTC status were performed by three research technicians who collectively had over 14 years of experience using the CellSearch system. Research technicians completed training by the manufacturer to identify positive and negative CTCs based on established criteria. Each sample was assigned to a primary reviewer for initial evaluation and a secondary reviewer for confirmatory evaluation. In any cases of ambiguity, final classification was discussed amongst the reviewers until a consensus was reached. All technicians were blinded to patient clinicopathologic data.

### 2.5. Outcomes

The primary outcome was rate of CTC-HER2 detection. Secondary outcomes included overall rate of CTC positivity, concordance rate of HER2 expression on primary tumor versus on CTCs, and overall survival. Overall survival was defined as the period from the date of the first determination of CTC positivity in positive patients and the first assessment overall in negative patients to the date of death. Patients who remained alive were censored on the date of last clinic follow up.

### 2.6. Statistical Analysis

Descriptive statistics (frequency, mean, median, percentages) were used to report patient and tumor characteristics. Wilcoxon rank-sum test was used to compare the distribution of continuous variables and Fisher’s exact test was used to compare the distribution of categorical variables. The Kaplan–Meier method and log-rank tests were used to estimate and compare overall survival by CTC-HER2 status. A *p*-value of less than 0.05 was considered statistically significant.

All statistical analyses were two-sided and performed using R version 4.3.1.

## 3. Results

### 3.1. Patient Selection

Of 1023 patients with invasive breast cancer, 174 had HER2-low breast cancer. Of these, 88 patients remained CTC negative throughout study enrollment, 17 patients had no available CTC data, and 30 patients were CTC positive but did not undergo CTC-HER2 status assessment due to lack of reagent availability at time of sample processing. The final study cohort consisted of 39 patients who had at least one positive CTC blood draw throughout treatment course and with available CTC-HER2 assessment(s). Figure 1 demonstrates the final study schema.

### 3.2. Baseline Patient Characteristics

Of the 39 HER2-low patients included, median age was 49 years (IQR 40.5–58.5) and median body mass index (BMI) was 29.9 kg/m^2^ (IQR 25.3–34.2) (Table 1). Race and ethnicity consisted of 66.7% (26) non-Hispanic White, 17.9% (7) non-Hispanic Black, 12.8% (5) Hispanic, and 2.6% (1) Asian. Majority of patients had clinical stage III-IV (26, 66.7%), node-positive (29, 76.3%), and ductal disease (27, 69.2%). Up to 59% (23) of patients had IBC. A total of 56.4% (22) were estrogen receptor (ER)-positive and 51.3% (20) were progesterone receptor (PR)-positive.

### 3.3. Treatment Patterns

In total, 74.4% (29) of patients received neoadjuvant chemotherapy and 76.9% (30) underwent surgical resection. Surgery consisted of modified radical mastectomy (15, 50.0%), segmental mastectomy (7, 23.3%), total mastectomy (4, 13.3%), skin-sparing mastectomy (3, 10.0%), and nipple-sparing mastectomy (1, 3.3%) (Table 2). Rate of pCR was 17.2%.

### 3.4. Rates of CTC and CTC-HER2 Positivity

A total of 95 blood draws were performed with a median of two blood draws per patient (IQR 2–3). Of these, 56 samples underwent CTC-HER2 assessment. The overall rate of CTC positivity was 43.9% (N = 69/157) among all patients with HER2-low disease and available CTC data. Among patients with one or more CTCs, rate of CTC-HER2 detection was 53.8% (N = 21/39) (Table 1). There were no significant differences in patient and tumor characteristics by CTC-HER2 status (Table 1). CTC positivity and CTC-HER2 positivity demonstrated dynamic fluctuations throughout treatment course (Table 3). Figure 2 demonstrates swimmer plots of CTC and CTC-HER2 status throughout the study period. Among patients with strong primary tumor HER2 expression (IHC 3+), concordance rate of HER2 expression on CTCs was 72.7% (N = 32/44) (Table 4). Among patients with HER2 negative primary tumor, discordance rate of HER2 expression on CTCs was 56.6% (N = 30/53).

### 3.5. Overall Survival by CTC-HER2 Expression

Patients who remained CTC-HER2 negative throughout serial blood draws demonstrated increased overall survival compared to those who were CTC-HER2 positive at any timepoint, although this was not statistically significant (Figure 3). The CTC-HER2 negative cohort had an estimated 5-year overall survival of 88.9% (95% CI 75.5–100.0) compared to 59.8% (95% CI: 41.5–86.2) for the CTC-HER2 positive cohort (HR for positive vs. negative = 3.30, 95% CI: 0.70–15.54). When overall survival was further stratified by primary tumor HER2 IHC score and CTC-HER2 status, patients with no HER2 expression on detectable CTCs demonstrated a trend toward increased overall survival compared to those with CTC-HER2 expression, regardless of whether the primary tumor IHC score was 1+ or 2+ (Figure 4).

## 4. Discussion

In this prospective exploratory cohort study of patients with HER2-low disease, a high proportion of patients demonstrated CTC-HER2 positivity despite having low expression of HER2 on their primary breast tumor. This discrepancy in HER2 expression between circulating tumor cells and the primary tumor highlights the variability in real time HER2 expression, even within the same patient. Importantly, our findings support the potential use of HER2 expression on CTCs as a biomarker to personalize cancer treatment in future prospective studies, particularly for patients who may be candidates for HER2-targeted therapies.

To our knowledge, this is the first study to evaluate CTC-HER2 expression exclusively among patients with HER2-low breast cancer. As these patients by definition exhibit weak to moderate HER2 expression in more than 10% of their primary tumor cells via IHC, accurate classification of HER2 status is imperative for informing treatment selection. Our results demonstrate that evaluation of HER2 status remains challenging due to its heterogeneous expression. Even within the same patient, we found fluctuations in HER2 receptor status between the primary breast tumor, metastatic site(s), and circulating tumor cells. These changes were observed both *within* and *across* different timepoints throughout treatment course. Furthermore, HER2 expression did not correlate proportionally with that expressed in the primary tumor. For instance, patients with HER2-negative breast cancer expressed comparable rates of CTC-HER2 compared to patients with HER2-low breast cancer. This finding again highlights the heterogenous nature of HER2 expression and the need to more accurately determine HER2 status in real time.

It is notable that over 50% of patients with HER2-low breast cancer had detectable CTC-HER2 throughout treatment course in this selected study cohort (N = 39). Furthermore, in the setting of no HER2-targeted therapy receipt, patients in the CTC-HER2 positive cohort exhibited a trend toward worse overall survival compared to those in the CTC-HER2 negative cohort, though this finding was not statistically significant. These findings highlight two key implications—(1) half of the study cohort may have been candidates for HER2-directed therapies and (2) CTC-HER2 directed therapy selection may be important for future exploratory prognostic analyses. Given the small sample size and exploratory nature of our study, these findings warrant further validation in larger patient populations. Indeed, studies focused on the HER2-negative population are currently assessing the clinical utility of treatment selection based on CTC-HER2 status. In the DETECT studies, CTC-HER2 status was assessed in over 1100 patients with metastatic HER2-negative breast cancer [9,17]. Presence of one or more CTC with strong HER2 staining was associated with worse overall survival compared to patients with negative or moderate HER2 staining [9]. However, this relationship did not remain significant on multivariable analysis. As a continuation of its parent study, the open label randomized DETECT III study evaluated the clinical benefit of CTC phenotyping in directing therapy selection [18]. Patients with HER2-negative metastatic breast cancer but HER2-positive CTCs were randomized to receive standard of care alone versus standard of care with lapatinib, a tyrosine kinase inhibitor targeting HER2 and epidermal growth factor receptor (EGFR). Patients receiving the HER2-directed therapy experienced a significantly longer median survival (20.5 months versus 9.1 months) compared to those receiving standard of care therapy on multivariable analysis. These pivotal findings from the DETECT III study form the basis for similar applications of CTC-HER2-guided treatment selection in patients with HER2-low breast cancer.

Given high rates of receptor discordance, current National Comprehensive Cancer Network (NCCN) and American Society of Clinical Oncology (ASCO) guidelines recommend repeat tissue sampling at metastatic site(s) to confirm biomarker status prior to treatment initiation in metastatic breast cancer [19,20]. However, adoption of this practice in real world settings is variable. Reported barriers to implementation include difficulty in accessing metastatic site(s) percutaneously, cost and invasiveness of undergoing another procedure, and patient preference [21,22,23]. As such, treatment of the metastatic disease is often dictated by the receptor status of the primary tumor. Circulating tumor cell enumeration and characterization represent a feasible, non-invasive solution to assess tumor biology in real time. In particular, CTC assessments more comprehensively capture the spatial and temporal heterogeneity of a patient’s tumor burden, as opposed to tissue biopsies which only sample one metastatic lesion. Furthermore, as CTCs are often collected alongside routine bloodwork, assessments can be easily incorporated into clinical practice and performed serially to track evolution of biomarker expression before, during, and after treatments. In addition to HER2, monitoring of other receptors such as ER, PR, and PD-L1 may help further delineate tumor phenotypes. For instance, characterization of PD-L1 status on CTCs have also revealed high discordance rates [24,25,26]. Studies have demonstrated that identifying CTC-PD-L1 cells may not only determine patient subsets more likely to respond to immunotherapy but also serve as a prognostic marker [24,25].

As with any study, our results should be interpreted in the setting of its limitations. For one, perhaps the biggest limitation to characterizing CTCs is that patients need to be CTC positive. Thus, for patients with early-stage disease, lower tumor burden, or disease biology with lower shedding rates, use of liquid biopsy to assess HER2 status may not be feasible. Second, there is currently no standardized definition for HER2-positive CTCs. Available studies use varying thresholds for positivity where some require at least 50% of CTCs to express HER2 while others consider presence of one CTC-HER2 as adequate [6,7,8,9,11]. We defined any patient with one or more CTC-HER2 as positive as these patients have demonstrated the potential for alterations in protein expression. Third, given that the CellSearch system detects only EpCAM-positive CTCs, our findings may underestimate the actual CTC population present since cells that undergo epithelial to mesenchymal transition may downregulate EpCAM expression. Given that cells with low EpCAM expression may not be captured or identified, non-detection by the CellSearch system is not conclusive for absence of CTCs and/or HER2-expressing CTCs. Fourth, this study was conducted at a comprehensive cancer center and may have limited generalizability. Furthermore, given that our institution has an established multidisciplinary inflammatory breast cancer clinic, a high proportion of our study cohort consisted of IBC patients. It is worth noting that our study includes a heterogenous cohort of patients with invasive breast cancer across different subtypes, disease burden, and receipt of local regional therapies, all of which may impact the degree of CTC shedding and survival analysis by CTC-HER2 status. Furthermore, given the small sample size, we were unable to conduct multivariable analysis. Nonetheless, this is the first study to evaluate CTC-HER2 status among patients with HER2-low breast cancer and future studies should aim to corroborate these findings in larger, more diverse, patient cohorts.

This study highlights the role of CTC characterization in providing real-time, non-invasive assessments of tumor phenotype over time. Among patients with HER2-low breast cancer, HER2 expression was highly dynamic with up to 50% of patients expressing CTC-HER2 cells at any given timepoint throughout treatment course in this study cohort. Accurate determination of HER2 status holds important implications for targeted tumor treatment. Specifically, it allows for identification of two distinct patient populations—(1) those with CTC-HER2 expression who may be undertreated and may benefit from HER2-targeted therapy and (2) those with loss of HER2 expression where further treatment with anti-HER2 may be ineffective. Given that this study was a small exploratory study, the clinical benefit of HER2-directed therapies in these two populations will require prospective, randomized trials. Beyond CTCs, HER2 assessments may be extended to a genomic, transcriptomic, and proteomic level. For instance, circulating tumor DNA, fragments of genomic material released by tumor cells into the bloodstream, may be sequenced to track mutations in real time [27,28,29]. For instance, Fusco and colleagues highlight the utility of tracking ESR1 mutations to identify patients with resistance to endocrine therapy in metastatic breast cancer. This approach may be applied similarly to track HER2 specific ctDNA mutations. Studies have also examined these blood-based markers using single-cell resolution analysis [30]. Furthermore, at a proteomics level, future development of automated algorithms for HER2 quantification on CTCs may reduce implicit bias and increase consistency in scoring. In the future, we envision a comprehensive liquid biopsy approach that incorporates multiple circulating biomarkers to more accurately classify receptor status among patients with breast cancer and guide treatment selection, with the ultimate goal of improving survival outcomes.

## 5. Conclusions

Patients with invasive HER2-low breast cancer demonstrate a high rate of CTC-HER2 expression despite low HER2 expression within the primary breast tumor. Characterization of HER2 levels on CTCs may serve as a useful biomarker to track intra- and intertumoral heterogeneity of HER2 expression. Findings from this study support future investigations of CTC-HER2 status in personalized therapy selection among breast cancer patients.

## Figures and Tables

**Figure 1:**
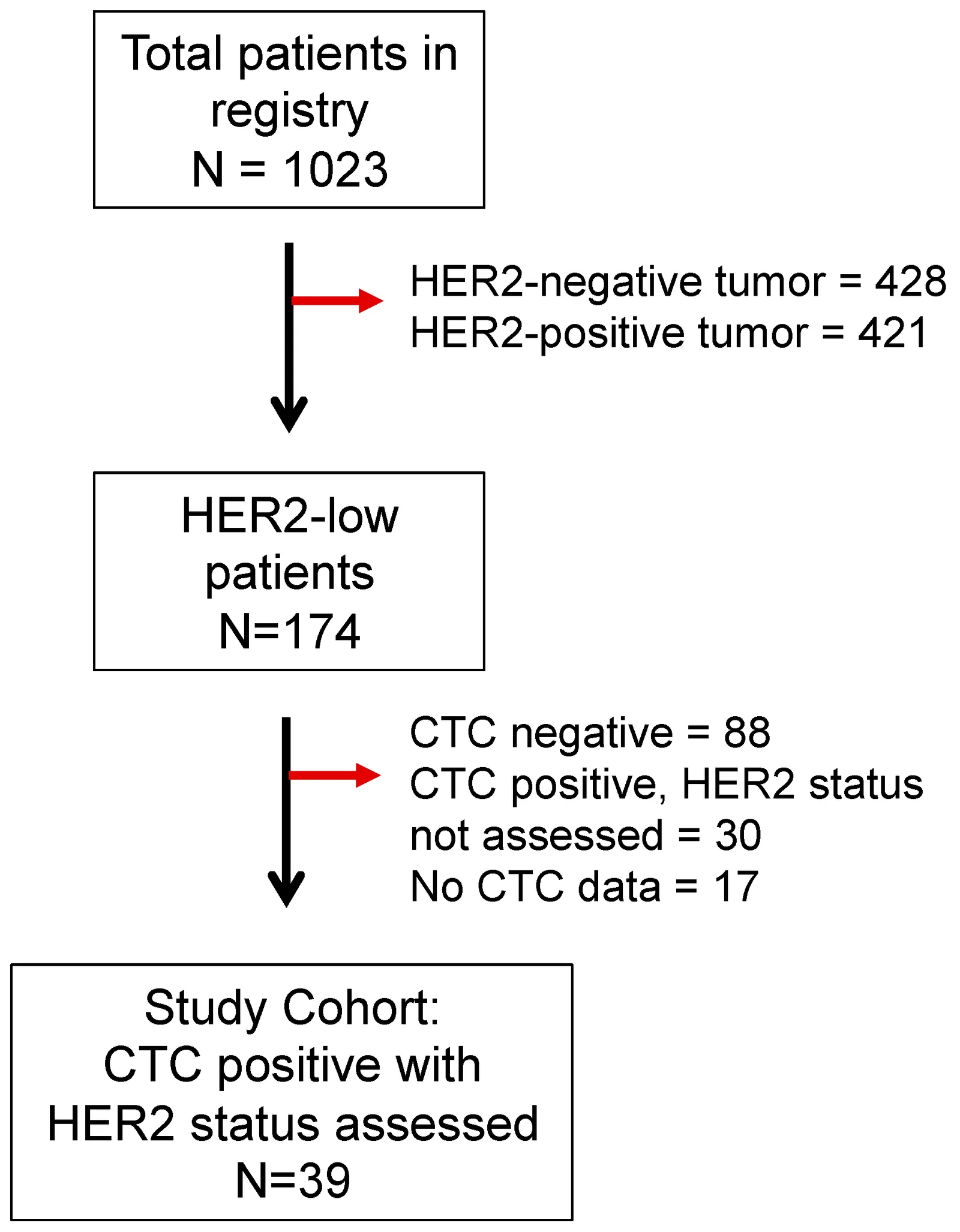
Study Schema.

**Figure 2:**
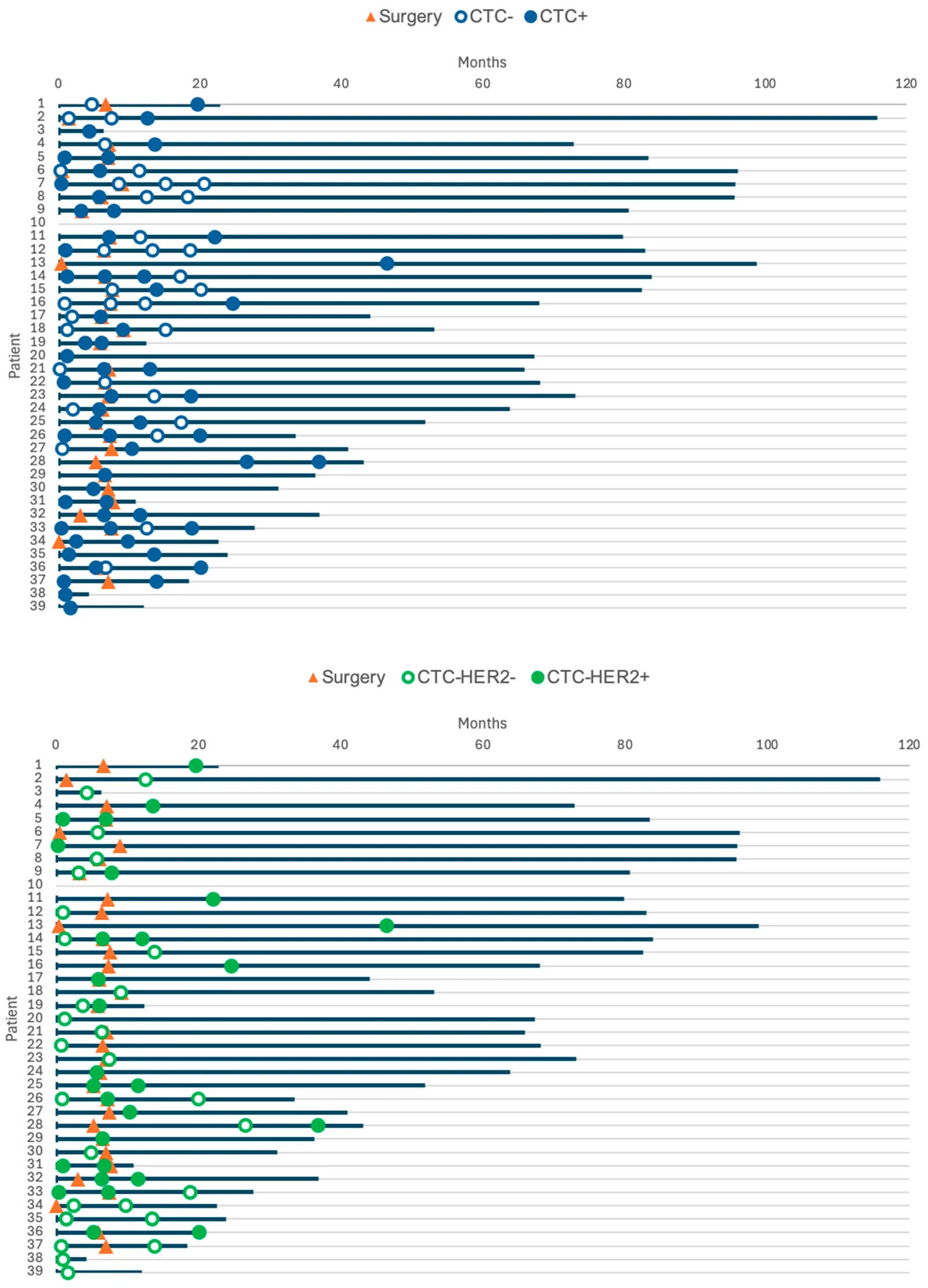
Swimmer Plots of CTC Status and CTC-HER2 Status Over Time. Of note, patient 10 was excluded due to long period of follow up.

**Figure 3:**
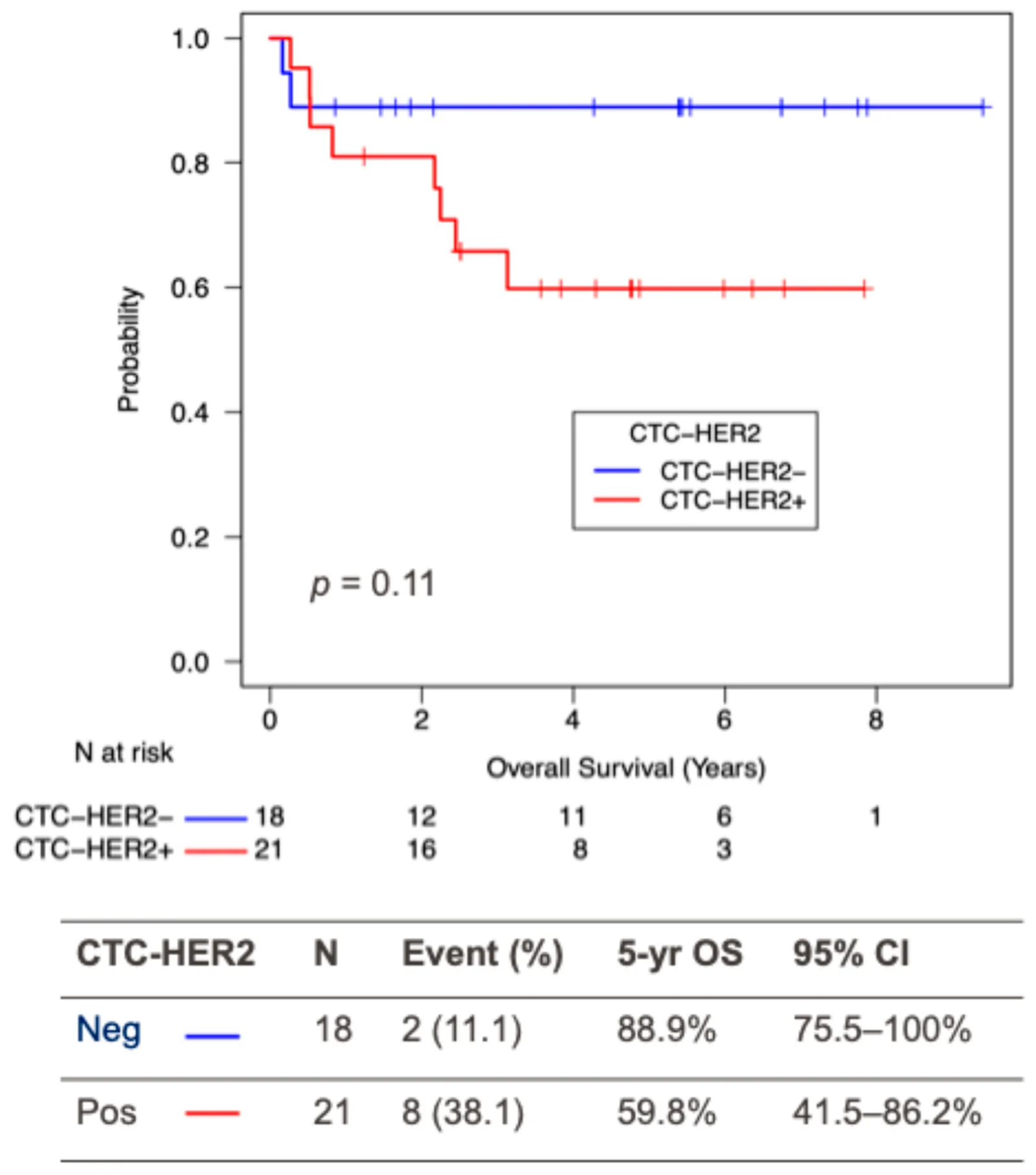
Overall Survival by CTC-HER2 Status.

**Figure 4:**
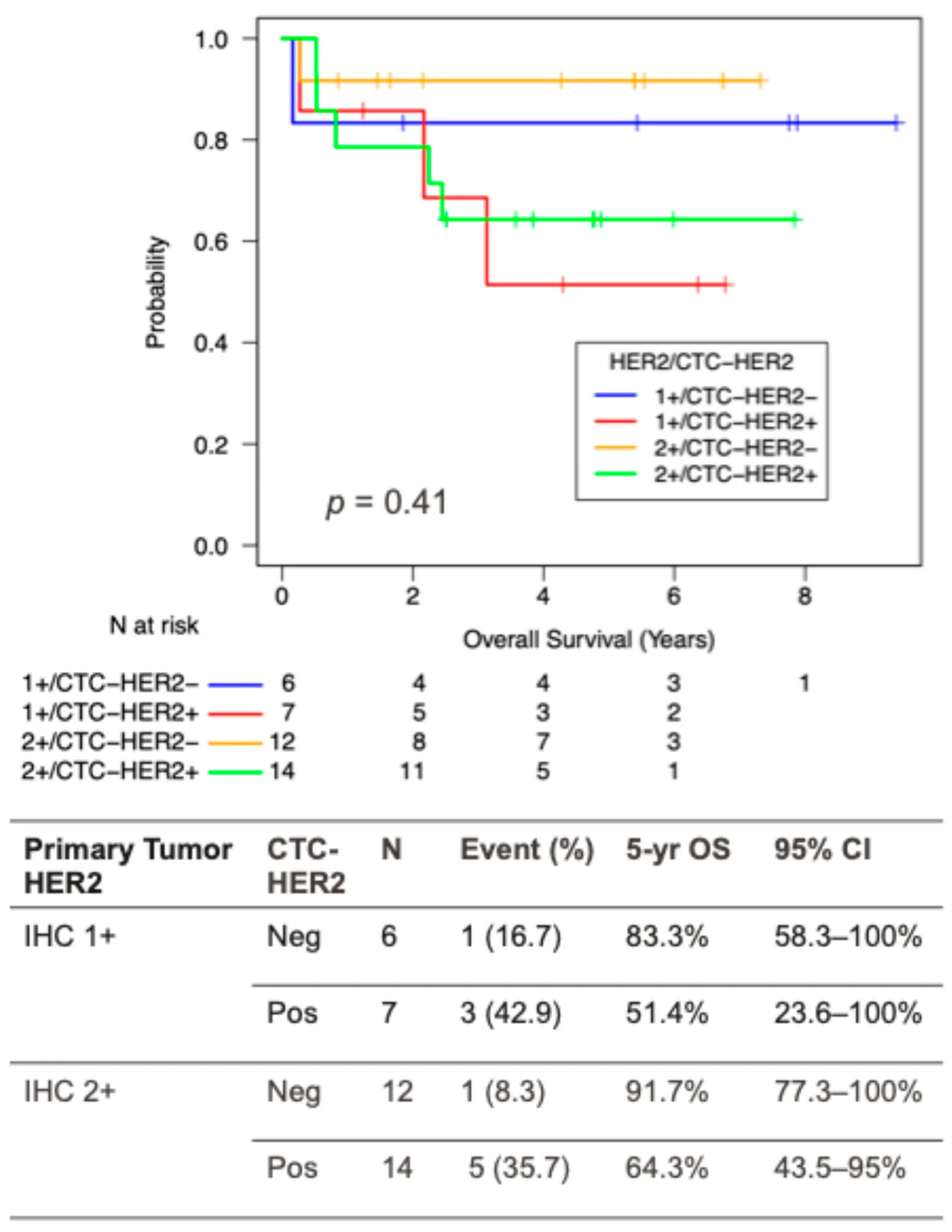
Overall Survival Stratified by Primary Tumor IHC Level and CTC-HER2 status.

**Table 1:** Baseline Patient Characteristics.

Variable	Total N (%), N = 39	CTC-HER2 Neg (n = 18)	CTC-HER2 Pos (n = 21)	*p*-Value
Age, median (IQR)	49.0 years (40.5–58.5)	52.0 years (43.0–58.8)	46.0 years (39.0–58.0)	0.61
BMI, median (IQR)	29.9 kg/m^2^ (25.3–34.2)	29.7 kg/m^2^ (24.5–35.3)	30.0 kg/m^2^ (26.1–32.1)	0.99
Missing	3	1	2	
Race and ethnicity				0.13
NH White	26 (66.7%)	9 (50.0%)	17 (81.0%)	
NH Black	7 (17.9%)	5 (27.8%)	2 (9.5%)	
Hispanic	5 (12.8%)	3 (16.7%)	2 (9.5%)	
Asian	1 (2.6%)	1 (5.6%)	0 (0.0%)	
AJCC cStage (8th Ed.)				0.65
I	4 (10.3%)	3 (16.7%)	1 (4.8%)	
II	9 (23.1%)	3 (16.7%)	6 (28.6%)	
III	15 (38.5%)	6 (33.3%)	9 (42.9%)	
IV	11 (28.2%)	6 (33.3%)	5 (23.8%)	
Nodal status				0.71
N0	9 (23.7%)	4 (23.5%)	5 (23.8%)	
N1	11 (28.9%)	4 (23.5%)	7 (33.3%)	
N2	4 (10.5%)	1 (5.9%)	3 (14.3%)	
N3	14 (36.8%)	8 (47.1%)	6 (28.6%)	
Missing	1	1	0	
Inflammatory BC	23 (59.0%)	10 (55.6%)	13 (61.9%)	0.75
ER+	22 (56.4%)	10 (55.6%)	12 (57.1%)	1.00
PR+	20 (51.3%)	10 (55.6%)	10 (47.6%)	0.75
HER2 IHC				1.00
1+	13 (33.3%)	6 (33.3%)	7 (33.3%)	
2+	26 (66.7%)	12 (66.7%)	14 (66.7%)	
Histology				0.35
Ductal	27 (69.2%)	12 (66.7%)	15 (71.4%)	
Lobular	2 (5.1%)	2 (11.1%)	0 (0.0%)	
Other	10 (25.6%)	4 (22.2%)	6 (28.6%)	
Grade				0.19
2	17 (43.6%)	10 (58.8%)	7 (33.3%)	
3	21 (53.8%)	7 (41.2%)	14 (66.7%)	
Missing	1	1	0	

**Table 2:** Treatment Patterns.

Variable	Total (N = 39, %)	CTC-HER2 Neg (n = 18, %)	CTC-HER2 Pos (n = 21, %)	*p*-Value
Received NAC	29 (74.4%)	12 (66.7%)	17 (81.0)	0.46
Surgery	30 (76.9%)	12 (66.7%)	18 (85.7%)	0.16
Modified radical mastectomy	15 (50.0%)	3 (25.0%)	12 (66.7%)	
Segmental mastectomy	7 (23.3%)	4 (33.3%)	3 (16.7%)	
Total mastectomy	4 (13.3%)	3 (25.0%	1 (5.6%)	
Nipple-sparing mastectomy	1 (3.3%)	0 (0.0%)	1 (5.6%)	
Skin-sparing mastectomy	3 (10.0%)	2 (16.7%)	1 (5.6%)	
None	9	6	3	
Pathologic complete response	5/29 (17.2%)	3/12 (25.0%)	2/17 (11.8%)	0.35
Lymphovascular invasion	10 (35.7%)	3 (30.0%)	7 (38.9%)	0.70
Missing	11	8	3	

**Table 3:** Rates of CTC and CTC-HER2 Positivity Throughout Treatment Course.

		Timepoint						
Detection Rate		Baseline	During NST	Post-NST/Pre-sx	3–6 Months Post-Op	6–12 Months Post-Op	>1 Year Post-Op	Total # of BDs
**CTC+**	N *	14/21	4/6	16/25	10/20	8/15	5/5	92
	**%**	66.7%	66.7%	64.0%	50.0%	53.3%	100.0%	
	**95% CI**	43.0–85.4%	22.2–95.7%	42.5–82.0%	27.2–72.8%	26.6–78.7%	47.8–100%	
**CTC-HER2+**	N *	4/14	1/4	11/15	5/8	3/7	4/5	53
	**%**	28.6%	25.0%	73.3%	62.5%	42.9%	80.0%	
	**95% CI**	8.4–58.1%	0.6–80.6%	44.9–92.2%	24.5–91.5%	9.9–81.6%	28.4–99.5%	

* N = 6 timepoints excluded as CTC (N = 3) and CTC-HER2 (N = 3) assessments occurred within 1-month post-surgery. # = number, BD = blood draw.

**Table 4:** Rates of HER2 Expression on Primary Tumor versus CTCs.

Primary Tumor HER2 Expression	IHC	FISH	Rate of CTC-HER2 Positivity	95% CI	*p*-Value
HER2-negative	0	n/a	30/53 (56.6%)	42.3–70.2%	0.149
HER2-low	1+	—	21/39 (53.8%)	37.2–69.9%	
	2+	Neg			
HER2-positive	3+	—	32/44 (72.7%)	57.2–85.0%	

n/a = not applicable.

## Data Availability

The supporting dataset for this study is available upon request from the corresponding author.

## Abbreviations

HER2 human epidermal growth factor receptor 2, IHC, FISH fluorescent in situ hybridization, CTC circulating tumor cells, IBC inflammatory breast cancer, AJCC American Joint Committee on Cancer, pCR pathologic complete response, EpCAM epithelial cell adhesion molecule, DAPI 4′,2-diamidino-2-phenylindole, BMI body mass index, ER estrogen receptor, PR progesterone receptor, EGFR epidermal growth factor receptor, NCCN National Comprehensive Cancer Network, ASCO American Society of Clinical Oncology.
